# Toward a possible trauma subtype of functional neurological disorder: impact on symptom severity and physical health

**DOI:** 10.3389/fpsyt.2022.1040911

**Published:** 2022-11-15

**Authors:** Sara Paredes-Echeverri, Andrew J. Guthrie, David L. Perez

**Affiliations:** ^1^Functional Neurological Disorder Research Group, Department of Neurology, Massachusetts General Hospital and Harvard Medical School, Boston, MA, United States; ^2^Functional Neurological Disorder Unit, Division of Cognitive Behavioral Neurology, Department of Neurology, Massachusetts General Hospital and Harvard Medical School, Boston, MA, United States; ^3^Division of Neuropsychiatry, Department of Psychiatry, Massachusetts General Hospital and Harvard Medical School, Boston, MA, United States

**Keywords:** functional neurological disorder, functional movement disorder, PTSD, symptom severity, physical health, childhood abuse, functional seizures, trauma

## Abstract

**Background:**

As a group, individuals with functional neurological disorder (FND) report an approximately 3-fold increase in adverse life experiences (ALEs) compared to healthy controls. In patients with FND, studies have identified a positive correlation between symptom severity and the magnitude of ALEs. While not all individuals with FND report ALEs, such findings raise the possibility of a trauma-subtype of FND.

**Objective:**

This study investigated if patients with FND, with or without probable post-traumatic stress disorder (PTSD) and/or significant childhood maltreatment, differed in their symptom severity and physical health.

**Materials and methods:**

Seventy-eight patients with FND were recruited (functional seizures, *n* = 34; functional movement disorder, *n* = 56). Participants completed self-report measures of symptom severity [Somatoform Dissociation Questionniare-20 (SDQ-20), Screening for Somatoform Disorders: Conversion Disorder subscale (SOMS:CD), Patient Health Questionniare-15 (PHQ-15)], physical health [Short Form Health Survey-36 (SF36-physical health)], childhood maltreatment [Childhood Trauma Questionnaire (CTQ)], and PTSD [PTSD Checklist-5 (PCL-5)]; a psychometric battery of other common predisposing vulnerabilities was also completed. To adjust for multiple comparisons, a Bonferroni correction was applied to all univariate analyses.

**Results:**

Patients with FND and probable PTSD (*n* = 33) vs. those without probable PTSD (*n* = 43) had statistically significant increased scores on all symptom severity measures – as well as decreased physical health scores. In secondary *post-hoc* regression analyses, these findings remained significant adjusting for age, sex, race, college education, and: pathological dissociation; alexithymia; attachment styles; personality characteristics; resilience scores; functional seizures subtype; or moderate-to-severe childhood abuse and neglect scores; SOMS:CD and SDQ-20 findings also held adjusting for depression and anxiety scores. In a separate set of analyses, patients with FND and moderate-to-severe childhood abuse (*n* = 46) vs. those without moderate-to-severe childhood abuse (*n* = 32) showed statistically significant increased SDQ-20 and PHQ-15 scores; in *post-hoc* regressions, these findings held adjusting for demographic and other variables. Stratification by childhood neglect did not relate to symptom severity or physical health scores.

**Conclusion:**

This study provides support for a possible trauma-subtype of FND. Future research should investigate the neurobiological and treatment relevance of a FND trauma-subtype, as well as continuing to delineate clinical characteristics and mechanisms in individuals with FND that lack a history of ALEs.

## Introduction

Functional neurological disorder (FND) is a prevalent neuropsychiatric condition characterized by distressing motor (e.g., limb weakness, tremors, seizures, etc.), sensory, and/or cognitive symptoms that are diagnosed based on positive features incongruent with other recognized pathology (1). Since early formulations of FND, including outdated terms such as “Hysteria” or “Conversion Disorder,” a range of traumatic events were identified as important predisposing vulnerabilities and/or acute precipitants for developing functional neurological symptoms. In the early 1900’s, Oppenheim described the condition as a “Traumatic Neurosis” that happened to military men, and others thought of the condition as related to post-traumatic stress disorder (PTSD) (2, 3). It is now well-documented that patients with FND compared to healthy controls have an approximately 3-fold greater occurrence of adverse life experiences (ALEs), including childhood maltreatment and other stressful life events across the lifespan (4). However, the 5th Edition of the Diagnostic and Statistical Manual of Mental Disorders (DSM-5) appropriately removed the requirement for a proximal stressor to make the diagnosis of FND—since not all patients endorse ALEs (5). Thus, questions remain regarding how to contextualize the role of ALEs in our modern-day conceptualization of FND (6).

Despite the lack of diagnostic specificity, several independent cohort studies reported positive associations between the magnitude of previously experienced ALEs and functional neurological symptom severity (7–12). For example, in a cohort of 54 patients with mixed FND – childhood physical abuse was associated with a larger number of distinct functional neurological symptoms; furthermore, endorsing multiple childhood traumatic experiences positively correlated with patient-reported symptom severity (9). In individuals with functional seizures (FND-seiz), those reporting childhood sexual abuse had earlier onset and more severe seizures (10). Individuals with FND and concurrent PTSD symptoms also endorsed more somatic symptoms compared to those with FND but without prominent PTSD symptoms (13). Additionally, a large-scale systematic review and meta-analysis identified that childhood abuse was associated with earlier symptom onset across the spectrum of FND (14). There is also some initial data to suggest that patients with FND and childhood maltreatment are associated with worse clinical outcomes (15, 16). Taken together, these findings highlight the clinical relevance of childhood ALEs and PTSD symptoms in FND when present—raising the possibility for a trauma-subtype of FND (17).

A potential trauma subtype—representing a subgroup of patients with the same clinical diagnosis but with additional distinguishing features—is not unique to FND. Emerging data suggests that a range of neuropsychiatric conditions across the spectrum of traditionally conceptualized psychiatric and neurological conditions may potentially show characteristics of a clinically-salient “trauma subtype” (17, 18). For example, literature in individuals with mood, anxiety and substance use disorders with childhood maltreatment highlights a propensity for increased symptom severity, comorbidity, suicidality, and worse treatment response compared to those without childhood maltreatment and the same psychiatric conditions (17). In the neurological literature, there is some evidence to support that the frequency and course of patients with headaches is worse in those who have experienced ALEs—particularly individuals with a history of childhood maltreatment (19–21). In adult men with Tourette Syndrome, a recent study also found an association between adverse childhood experiences and lifetime tic severity (22).

In this cohort study, we aimed to further investigate the possibility of a trauma-subtype of FND—with a focus on potential relationships to symptom severity and physical health. We stratified our mixed FND cohort based on the presence or absence of probable PTSD, and separately based on the presence or absence of endorsed childhood maltreatment (abuse and neglect considered separately) (23). Together, these complementary approaches allowed us to consider the potential implications of childhood maltreatment and/or the ongoing subjective impact of ALE-related psychopathology in potentially delineating FND subgroups (23). We hypothesized that patients with either probable PTSD and/or moderate-to-severe childhood maltreatment scores would demonstrate increased symptom severity and worse physical health scores.

## Materials and methods

### Participants

Seventy-eight adults diagnosed with FND (53 females; 25 males; mean age 42.5 ± 13.9 years old; mean illness duration 4.0 ± 4.9 years) based on “rule in” examination signs and semiological features were prospectively recruited for study participation following an outpatient evaluation at the Massachusetts General Hospital FND Unit from 2014 to 2020 (see Supplementary Table 1 for additional details) (1). Importantly, given that patients commonly present with mixed functional neurological symptoms, we took a transdiagnostic approach across the spectrum of functional movement disorder (FND-movt) and FND-seiz (24, 25). Inclusion criteria were individuals 18 years of age or older with clinically-established FND-movt [*n* = 56; including functional weakness (*n* = 24), tremor (*n* = 23), gait (*n* = 22), jerks (*n* = 2), dystonia (*n* = 4), and speech difficulties (*n* = 5)] and/or FND-seiz [*n* = 34; composed of probable (*n* = 2), clinically-established (*n* = 2), or video-electroencephalography documented functional seizures (*n* = 30)] (26). FND diagnoses were not mutually exclusive, as 12 individuals had both FND-movt and FND-seiz. Patients with major neurologic comorbidities (*n* = 11, including 4 with epileptic seizures) were included. Exclusion criteria were illiteracy, history of mania or psychosis, active suicidality, and current illicit drug abuse or alcohol dependence. All participants provided written informed consent as approved by the institutional review board of Mass General Brigham. Note, psychometric findings from this cohort, unrelated to the present set of analyses, have been published elsewhere (7, 27–29).

### Post-traumatic stress disorder and childhood maltreatment self-report measures

The PTSD Checklist for DSM-5 (PCL-5) was used as one approach to stratify the FND cohort (30, 31). The PCL-5 has 20-items on the experience of PTSD symptoms in the past month, each rated “Not at all” (scored 0) to “Extremely” (scored 4), with a total score of ≥33 indicative of probable PTSD (30, 31). Individuals meeting current probable PTSD criteria were included in the “FND-PTSD_high” subgroup and all others placed in the “FND-PTSD_low” subgroup. Separately, the abuse and neglect subscale scores of the Childhood Trauma Questionnaire (CTQ) were used as alternative ways of dichotomizing the FND cohort (32). The 25-item CTQ assesses for experiences of abuse and neglect during childhood and adolescence. The cut-off values for moderate-to-severe subscale scores are as follows: ≥13 for emotional abuse, ≥10 for physical abuse, ≥8 for sexual abuse, ≥15 for emotional neglect, and ≥10 for physical neglect (33). To determine our “FND-Abuse_high” and “FND-Neglect_high” subgroups, any of the three abuse subscales or two neglect scales had to be equal to or greater than cut-off scores, respectively. We grouped physical, sexual, and emotional abuse scores together since they were strongly correlated, as were physical and emotional neglect scores.

### Symptom severity and physical health self-report measures

Participants completed three symptom severity scales: the Somatoform Dissociation Questionnaire-20 (SDQ-20)—a 20-item questionnaire of functional neurological symptoms (e.g., seizures, paralysis, etc.) in the past year with each item rated on a 5-point Likert-type scale from “Not at all” (scored 1) to “Extremely” (scored 5) (34, 35); the Screening for Somatoform Symptoms-7 subscale for Conversion Disorder (SOMS:CD)—a 14-item questionnaire of functional neurological symptoms in the past week, with each item rated on a 5-point Likert scale from “Not at all” (scored 0) to “Severe” (scored 4) (36); and the Patient Health Questionnaire-15 (PHQ-15)—a 15-item measure of functional somatic symptoms (e.g., pain, fatigue) within the past 4 weeks, with each item rated from “Not Bothered at All” (scored 0) to “Bothered A Lot” (scored 2) (37). The physical health subscales of the Short Form Health Survey-36 were used to derive the physical health component score (SF36-physical health)—a measure of overall physical wellness and the extent to which physical symptoms limit an individual’s abilities to work and/or perform regular daily activities, with lower scores indicating greater disability (38).

### Additional self-report measures

To allow for *post-hoc* secondary analyses controlling for other relevant predisposing vulnerabilities, the following psychometric scales were also collected: Beck Depression Inventory-II (BDI)—a 21-item scale measuring depression symptom severity within the past 2 weeks, with each item scored from 0 to 3; the 20-items of the trait anxiety subscale of the Spielberger State-Trait Anxiety Inventory (STAI-trait), with each item scored in a 4-point scale ranging from “Almost Never” to “Almost Always”; the Dissociative Experiences Scale (DES)—a 28-item measure of dissociative experiences in daily life rated from 0 to 100%, with a cut-off mean value of ≥30% for pathological dissociation; Toronto Alexithymia Scale (TAS)—a 20-item measure of difficulty identifying/describing feelings and externally-oriented thinking, with each item scored on a 5-point Likert scale; Relationships Scales Questionnaire (RSQ)—a 30-item dimensional measure of adult attachment styles with each item scored on a 5-point Likert scale; NEO Five Factor Inventory-3 (NEO-FFI-3)—a 60-item measure characterizing the “Big Five” personality traits, with each item scored on a 5-point Likert-type scale from “Strongly Disagree” to “Strongly Agree”; and the Connor-Davidson Resilience Scale (CDRS)—a 25-item item scale accessing stress coping tendencies used in the past month, with each item scored on a 5-point Likert scale.

### Data analysis

STATA-17 BE software (StataCorp LLC, College Station, Texas, USA) was used to run all statistical analyses, and missing data (*n* = 2) were omitted from analyses. We ran the Shapiro–Wilk test (command *swilk)* to determine the distribution of outcome measurements. A student *t*-test (command *ttest*) was used for normally distributed data and the Mann–Whitney Wilcoxon rank sum test (command *ranksum*) for non-parametric data, to investigate potential statistically significant differences in symptom severity (SDQ-20, SOMS:CD, and PHQ-15) and/or SF36-physical health scores between our stratified FND subgroups: (1) FND-PTSD_high vs. FND-PTSD_low; (2) FND-Abuse_high vs. FND-Abuse_low; (3) FND-Neglect_high vs. FND-Neglect_low. A Bonferroni correction for multiple comparisons was applied to the alpha value of 0.05 across all univariate statistics; only findings that remained significant entered secondary (*post-hoc*) linear regression analyses.

For all statistically significant univariate findings corrected for multiple comparisons, we conducted separate *post-hoc* linear regressions with the command *regress* always adjusting in blocks for the demographic variables of age, sex, white race (yes/no) and college graduate (yes/no), and other variables as follows: (1) only the previously mentioned demographic variables; (2) depression (BDI) and anxiety (STAI-trait) scores; (3) pathological dissociation (DES ≥ 30% yes/no); (4) TAS total score; (5) RSQ subscale scores for dismissing, fearful, preoccupied, and secure attachment styles; (6) NEO agreeableness, conscientiousness, extraversion, neuroticism, and openness personality trait scores; (7) CDRS total score; (8) FND-seiz (yes/no); (9) moderate-to-severe CTQ abuse and neglect scores (yes/no) in FND-PTSD_high analyses, or FND-PTSD_high and moderate-to-severe CTQ neglect (yes/no) if predicting FND-Abuse_high. For all regressions, variance inflation factor (*estat vif*) was used to look for multicollinearity amongst the independent variables.

## Results

See Table 1 for the breakdown of demographic variables and psychometric scores for FND-PTSD_high (*n* = 33) vs. FND-PTSD_low (*n* = 43), FND-Abuse_high (*n* = 46) vs. FND-Abuse_low (*n* = 32), and FND-Neglect_high (*n* = 33) vs. FND-Neglect_low (*n* = 45).

**TABLE 1:** Demographic and clinical characteristics of stratified functional neurological disorder subgroups.

	FND-PTSD_high (*N* = 33)		FND-PTSD_low (*N* = 43)		FND-Abuse_high (*N* = 46)		FND-Abuse_low (*N* = 32)		FND-Neglect_high (*N* = 33)		FND-Neglect_low (*N* = 45)	
						
	Mean or N	SD or %	Mean or N	SD or %	Mean or N	SD or %	Mean or N	SD or %	Mean or N	SD or %	Mean or N	SD or %
**Demographics**												
Age	43.4	(13.1)	41.1	(14.6)	44.2	(12.8)	39.9	(15.3)	44.2	(13.0)	41.2	(14.5)
Female sex	22	(66.7)	30	(69.8)	31	(67.4)	22	(68.8)	21	(63.6)	32	(71.1)
White race	25	(75.8)	40	(93.0)	38	(82.6)	29	(90.6)	29	(87.9)	38	(84.4)
College graduate	9	(27.3)	23	(53.5)	19	(41.3)	14	(43.8)	15	(45.5)	18	(40.0)
**FND subtype**												
FND-seiz	15.0	(45.5)	19.0	(44.2)	20.0	(43.5)	14.0	(43.8)	14.0	(42.4)	20.0	(44.4)
FND-movt	23.0	(69.7)	31.0	(72.1)	33.0	(71.7)	23.0	(71.9)	24.0	(72.7)	32.0	(71.1)
**Comorbidities**												
Major neurological comorbidities	5.0	(15.2)	5.0	(11.6)	5.0	(10.9)	6.0	(18.8)	5.0	(15.2)	6.0	(13.3)
**Psychometric measurements**												
BDI	28.6	(9.6)	13.8	(10.0)	23.4	(12.5)	14.8	(10.3)	20.7	(11.1)	19.3	(13.2)
STAI-trait	53.6	(8.7)	42.5	(9.7)	50.2	(10.3)	42.7	(10.1)	48.6	(9.1)	46.0	(11.9)
Pathological DES	16	(48.5)	3	(7.0)	15	(32.6)	4	(12.5)	10	(30.3)	9	(20.0)
TAS	59.6	(13.0)	54.3	(13.2)	58.7	(14.0)	53.8	(11.8)	59.0	(14.7)	55.0	(12.0)
RSQ: Dismissing	3.5	(0.6)	3.5	(0.5)	3.6	(0.5)	3.4	(0.6)	3.5	(0.5)	3.5	(0.6)
RSQ: Fearful	3.4	(1.1)	2.5	(0.9)	3.2	(1.1)	2.4	(0.9)	3.0	(1.1)	2.8	(1.1)
RSQ: Preoccupied	2.7	(0.8)	2.6	(0.8)	2.8	(0.8)	2.5	(0.6)	2.8	(0.8)	2.6	(0.8)
RSQ: Secure	2.9	(0.7)	3.1	(0.6)	2.9	(0.6)	3.1	(0.5)	2.9	(0.6)	3.0	(0.6)
NEO: Agreeableness	35.1	(5.2)	35.0	(5.2)	35.8	(5.5)	34.1	(4.6)	35.2	(6.2)	35.0	(4.4)
NEO: Conscientiousness	30.5	(8.1)	31.9	(6.3)	31.0	(7.4)	31.6	(6.9)	31.4	(6.9)	31.1	(7.3)
NEO: Extraversion	21.5	(6.1)	26.6	(6.9)	24.2	(7.6)	24.9	(6.2)	24.0	(7.5)	24.8	(6.7)
NEO: Neuroticism	31.4	(8.2)	23.1	(7.9)	28.7	(9.0)	23.8	(8.2)	26.6	(9.6)	26.7	(8.5)
NEO: Openness	27.0	(5.8)	26.1	(5.5)	27.4	(5.9)	25.2	(5.1)	27.3	(5.9)	25.9	(5.4)
CDRS	57.2	(15.2)	68.5	(14.1)	62.0	(16.1)	66.1	(14.5)	63.6	(16.2)	63.7	(15.2)

Demographic, FND subtype, comorbidities, and psychometric measurement breakdown of our sample per trauma subtypes determined by the cut-off scores of the PCL-5, and CTQ abuse and neglect subscales. In terms of missing data, one person did not complete their NEO or PCL-5; another also missed the PCL-5. BDI, Beck Depression Inventory II; CDRS, Connor-Davidson Resilience Scale; CTQ, Childhood Trauma Questionnaire; DES, Dissociative Experiences Scale; FND, Functional Neurological Disorder; FND-movt, Functional Movement Disorder; FND-seiz, Functional Seizures; NEO, NEO Five Factor Inventory-3; PCL-5, PTSD Checklist for DSM-5; PTSD, Post Traumatic Stress Disorder; RSQ, Relationship Scales Questionnaire; STAI-trait, Spielberger Trait Anxiety Inventory Subscale; SD, Standard Deviation; TAS, Toronto Alexithymia Scale; %, percent of the total subgroup sample.

The PHQ-15 and SF36-physical health scores were normally distributed, whereas the SOMS:CD and SDQ-20 scores were not. All symptom severity scores were significantly increased, and SF36-physical health scores significantly decreased, in patients with FND-PTSD_high vs. FND-PTSD_low. Additionally, patients with FND-Abuse_high vs. FND-Abuse_low showed statistically significant increases in SDQ-20 and PHQ-15 scores. There were no statistically significant differences in symptom severity or physical health scores in patients with FND-Neglect_high vs. FND-Neglect_low (see Table 2).

**TABLE 2:** Symptom severity and physical health scores in functional neurological disorder stratified by trauma subtypes.

	FND-PTSD_high (*N* = 33)		FND-PTSD_low (*N* = 43)			FND-Abuse_high (*N* = 46)		FND-Abuse_low (*N* = 32)			FND-Neglect_high (*N* = 33)		FND-Neglect_low (*N* = 45)		
								
	Mean	SD	Mean	SD	*P*-value	Mean	SD	Mean	SD	*P*-value	Mean	SD	Mean	SD	*P*-value
SDQ-20	39.1	10.8	28.6	7.5	<0.001*	35.8	11.2	28.9	7.7	0.001*	35.0	11.7	31.5	9.1	0.133
PHQ-15	16.0	4.3	11.8	4.6	<0.001*	15.2	4.8	11.3	4.3	<0.001*	14.2	4.9	13.2	5.0	0.390
SOMS:CD	12.4	7.0	6.3	5.5	<0.001*	9.9	7.4	7.2	5.9	0.147	8.2	7.2	9.2	6.7	0.417
SF36-PH	37.7	19.5	52.9	21.6	0.002*	42.4	21.4	52.2	21.7	0.051	45.3	21.8	47.2	22.2	0.708

The symptom severity measures are SDQ-20, PHQ-15, and SOMS:CD; physical health is measured with the SF36 physical component score. *P*-values with an asterisk (*) are statistically significant after Bonferroni correction. FND, Functional Neurological Disorder; PHQ-15, Participant Health Questionnaire; SD, Standard Deviation; SDQ-20, Somatoform Dissociation Questionnaire; SF36-PH, Short Form Health Survey-36 physical health component score; SOMS:CD, Screening for Somatoform Symptoms-Conversion Disorder Subscale.

In separate *post-hoc* linear regression analyses, FND-PTSD_high vs. FND-PTSD_low relationships to increased symptom severity and reduced SF36-physical health scores remained statistically significant adjusting for demographic variables and sequentially correcting for pathological dissociation; alexithymia; attachment styles; personality traits; resilience scores; FND-seiz; and for the other trauma scales. Only relationships between FND-PTSD_high and symptom severity as measured by the SDQ-20 and SOMS:CD also remained significant adjusting for depression and trait anxiety scores.

Additionally, in separate *post-hoc* linear regression analyses, FND-Abuse_high vs. FND-Abuse_low relationships to symptom severity (i.e., PHQ-15, SDQ-20) remained statistically significant adjusting serially for demographic and all other neuropsychiatric variables. There was no evidence of problematic collinearity across all regression analyses as measured by variance inflation factors. See Supplementary Tables 2–7 for a detailed description of all completed *post-hoc* analyses.

## Discussion

Our cohort study found that the FND-PTSD_high subgroup reported increased symptom severity and decreased physical health compared to those in the FND-PTSD_low subgroup; this finding remained statistically significant in *post-hoc* analyses adjusting for other variables except for when controlling for depression and anxiety scores in SF36-physical health or PHQ-15 related analyses. Furthermore, stratification based on the presence of moderate-to-severe childhood abuse also showed statistically significant increases in symptom severity in the FND-Abuse_high subgroup—findings that held for all *post-hoc* analyses. Stratification per childhood neglect did not relate to either patient-reported symptom severity or physical health scores. This data lends support to the possibility of a clinically-significant trauma subtype of FND (based on high comorbid PTSD symptoms and/or high childhood abuse burden).

Consideration of a trauma subtype of FND is consistent with emerging themes in neurobiological research in this population. In gray matter quantitative analyses in patients with FND, childhood maltreatment and ALEs more broadly are linked to insula, amygdala, hippocampus, putamen, and cerebellum structural alterations (12, 39, 40). Similarly, several resting-state functional magnetic resonance imaging (fMRI) studies identified that the connectivity profiles of nodes implicated in the pathophysiology of FND (i.e., insula, amygdala, temporoparietal junction, motor control areas) are impacted by the magnitude of early life adversity (41–43). For example, one study showed that the magnitude of childhood physical abuse severity related to how closely coupled the amygdala and insula were to the primary motor cortex; this study included a psychiatric control group matched for trauma burden—with the psychiatric control group not showing similar brain-trauma functional connectivity relationships (41). In a neuroimaging—genetics study, the magnitude of endorsed childhood trauma (CTQ total score) in patients with FND with a tryptophan gene polymorphism positively correlated with symptom severity; this genetic variant also impacted the connectivity between the amygdala and middle frontal gyrus (44). In addition to the neuroimaging literature, sexual trauma correlated with increased basal diurnal salivary cortisol in patients with FND-seiz, as well as a decreased cortisol and amylase response to a social stress test (45, 46). Lastly, a study found that a history of sexual trauma in patients with FND-seiz correlated with increased vigilance to a social threat during a Trier Social Stress Task (47). Overall, the literature points toward a clinical and neurobiologically distinct subtype of FND in the context traumatic experiences.

There are treatment implications for a potential framing of a trauma subtype of FND, most notably psychotherapeutic considerations (48). In the largest psychotherapy trial conducted to date, COgnitive behavioral therapy for adults with Dissociative non-Epileptic Seizures (CODES) trial, 368 patients with FND-seiz were randomized to cognitive behavioral therapy (CBT) plus standardized medical care (*n* = 186) vs. standardized medical care alone (*n* = 182) (49, 50). Although many secondary benefits were found in the CBT treatment arm, the study did not identify a statistically significant treatment effect on the primary outcome of seizure frequency at 12-months post-randomization. Since the publication of this landmark study, several research groups have advocated for the need to refine the pairing of specific psychotherapy modalities to a given patient with FND based on clinical formulation (e.g., what else is present alongside this patient’s functional neurological symptoms) (51–53). For example, there is evidence to suggest that patients with FND-seiz and PTSD may benefit from prolonged exposure (PE) therapy (54); other psychotherapy treatment modalities such as dialectical behavioral therapy (DBT), eye movement desensitization and reprocessing (EMDR), mindfulness-based psychotherapy (MBT), and acceptance and commitment therapy (ACT) have all been piloted in FND populations (55–58). Operationalizing a trauma-subtype of FND would facilitate increased recognition and research efforts to study how to best pair a given patient with FND to the psychotherapy modality they are most likely to benefit from. Defining this subgroup would also open new areas of therapeutic research inquiry, such as consideration of other forms of trauma-informed psychotherapy (e.g., sensorimotor psychotherapy) and the need for additional clinical trial research in treatment refractory individuals (e.g., testing the potential utility of ketamine infusions) (59, 60).

There are several gaps that remain with regards to the kind of data needed to adequately define a trauma subtype of FND. One of the most critical features to consider further is the nature of traumatic experiences and their impact on a given individual. In this study, the self-report of PTSD symptoms based on the PCL-5 with a cut-off indicative of probable PTSD drew significant results for the comparison of FND-PTSD_high vs. FND-PTSD_low in three symptom severity and one physical health measure. Alternatively, the CTQ abuse subscale measuring reported childhood sexual, physical and emotional abuse was used to divide individuals into FND-Abuse_high vs. FND-Abuse_low subgroups—with this stratification approach also leading to significant associations with two patient-reported symptom severity scores. The endorsement of active PTSD symptoms can be thought of as a proxy for the subjective current impact of past traumatic experiences. CTQ scores help quantify patient-reported childhood maltreatment regardless of concurrent PTSD symptoms. Additional work is needed to further understand if subjective ongoing relevance, subjective occurrence, or both are important factors in how to operationalize a trauma subtype of FND. Notably, large scale research has identified that the subjective occurrence of childhood maltreatment is a strong predictor of the development of psychopathology, irrespective of objective documentation of such experiences (23).

This study has several limitations, including the lack of a DSM-5 structured psychiatric interview and no lifetime measure of ALE severity. We also do not have information on the specific timing of ALEs, and as such we are unable to comment on the potential influence of critical developmental periods in linking childhood abuse to later life FND symptom severity. Other nuanced trauma-related factors such as escape potential require continued inquiry (61, 62), as well as a comprehensive investigation of potential mediators and moderators of relationships between ALEs, PTSD, symptom severity, and physical health in patients with FND. Discrete forms of childhood abuse (physical vs. sexual vs. emotional) may also be important considerations needing more research. Our study found that associations between the FND-PTSD_high subgroup and physical health or somatic symptom scores did not remain significant when adjusting for depression/anxiety scores, underscoring close associations between mood/anxiety, non-motor symptoms and physical health-related quality of life (63, 64). Additionally, while several independent FND cohorts showed positive associations between trauma burden and symptom severity (7–10, 12), one study of individuals with FND-seiz in Iran did not identify this association (65). This suggests that larger scales replication studies are needed, including consideration of sociocultural factors. Furthermore, it will be important to examine similarities and differences between a trauma subtype of FND and other psychological trauma-related clinical populations (66, 67). It also remains unclear what mechanisms link ALEs to FND symptom severity. Some considerations include overlapping brain–trauma and brain–symptom severity relationships, as well as possible shared predictive processing influences. For example, life experiences impact the repertoire of emotion and non-emotion concepts, and differences in emotion granularity and emotion category construction warrant future research as potential bridges between ALEs and the development/severity of FND symptoms (68). Lastly, prospective longitudinal studies are needed to determine if clinical outcomes reliably differ in patients with FND with or without a history of trauma.

In conclusion, this study lends support to a possible trauma-subtype of FND–across the transdiagnostic spectrum of FND-movt and FND-seiz. Additional research is needed to replicate and expand upon the findings of this study, including investigating if neural mechanisms and clinical outcomes are the same or different in patients with FND with or without a history of childhood maltreatment (or concurrently active PTSD symptoms).

## Data availability statement

The dataset presented in this article are not readily available because sharing of this data requires local IRB approval. However, on reasonable request by other researchers, anonymized raw data related to this research will be made available pending approval by the local IRB. Requests to access the dataset should be directed to DP, dlperez@nmr.mgh.harvard.edu.

## Ethics statement

The studies involving human participants were reviewed and approved by Mass General Brigham IRB, Massachusetts General Hospital. The patients/participants provided their written informed consent to participate in this study.

## Author contributions

All authors were involved in all stages of preparing this manuscript, including the literature review, performance of statistical analyses, drafting and editing the manuscript, and approved the submitted version.
