# Corrigendum: Toward a possible trauma subtype of functional neurological disorder: impact on symptom severity and physical health

**DOI:** 10.3389/fpsyt.2023.1192755

**Published:** 2023-04-18

**Authors:** Sara Paredes-Echeverri, Andrew J. Guthrie, David L. Perez

**Affiliations:** ^1^Functional Neurological Disorder Research Group, Department of Neurology, Massachusetts General Hospital and Harvard Medical School, Boston, MA, United States; ^2^Functional Neurological Disorder Unit, Division of Cognitive Behavioral Neurology, Department of Neurology, Massachusetts General Hospital and Harvard Medical School, Boston, MA, United States; ^3^Division of Neuropsychiatry, Department of Psychiatry, Massachusetts General Hospital and Harvard Medical School, Boston, MA, United States

**Keywords:** functional neurological disorder, functional movement disorder, PTSD, symptom severity, physical health, childhood abuse, functional seizures, trauma

In the published article, there was an error in Table 2 as published. The count of FND-PTSD_low is 43 and not 45. The corrected Table 2 and its caption “Symptom severity and physical health scores in functional neurological disorder stratified by trauma subtypes” appear below.

**Table 2:** Symptom severity and physical health scores in functional neurological disorder stratified by trauma subtypes.

	**FND-PTSD_high (*****N*** = **33)**		**FND-PTSD_low (*****N*** = **43)**		**p-value**	**FND-Abuse_high (*****N*** = **46)**		**FND-Abuse_low (*****N*** = **32)**		**p-value**	**FND-Neglect_high (*****N*** = **33)**		**FND-Neglect_low (*****N*** = **45)**		**p-value**
	**Mean**	**SD**	**Mean**	**SD**		**Mean**	**SD**	**Mean**	**SD**		**Mean**	**SD**	**Mean**	**SD**	
SDQ-20	39.1	10.8	28.6	7.5	< 0.001^*^	35.8	11.2	28.9	7.7	0.001^*^	35.0	11.7	31.5	9.1	0.133
PHQ-15	16.0	4.3	11.8	4.6	< 0.001^*^	15.2	4.8	11.3	4.3	< 0.001^*^	14.2	4.9	13.2	5.0	0.390
SOMS:CD	12.4	7.0	6.3	5.5	< 0.001^*^	9.9	7.4	7.2	5.9	0.147	8.2	7.2	9.2	6.7	0.417
SF36-PH	37.7	19.5	52.9	21.6	0.002^*^	42.4	21.4	52.2	21.7	0.051	45.3	21.8	47.2	22.2	0.708

The symptom severity measures are SDQ-20, PHQ-15, and SOMS:CD; physical health is measured with the SF36 physical component score. P-values with an asterisk (^*^) are statistically significant after Bonferroni correction. FND, Functional Neurological Disorder; PHQ-15, Participant Health Questionnaire; SD, Standard Deviation; SDQ-20, Somatoform Dissociation Questionnaire; SF36-PH, Short Form Health Survey-36 physical health component score; SOMS:CD, Screening for Somatoform Symptoms-Conversion Disorder Subscale.

The authors apologize for this error and state that this does not change the scientific conclusions of the article in any way. The original article has been updated.

