# Supplementary material for: Toward a possible trauma subtype of functional neurological disorder: impact on symptom severity and physical health
Source: Front Psychiatry. 2022 Nov 15;13:1040911. doi: 10.3389/fpsyt.2022.1040911 (PMC9706184; doi:10.3389/fpsyt.2022.1040911)
Supplement: Supplementary file 1 [file Data_Sheet_1.docx]

| Demographic and Clinical Characteristics of the Functional Neurological Disorder Cohort (N=78) | | |
| --- | --- | --- |
|  | **Mean or N** | **SD or %** |
| *Demographic* |  |  |
| Age | 42.5 | (13.9) |
| Female Sex | 53 | (67.9) |
| White Race | 67 | (85.9) |
| College Graduate | 33 | (42.3) |
| *FND Subtype* |  |  |
| Illness duration in years | 4.0 | (4.9) |
| FND-seiz | 34 | (43.6) |
| FND-movt | 56 | (71.8) |
| *Comorbidities* |  |  |
| Major Neurological Comorbidities | 11 | (14.1) |
| *Psychometric Measurements* |  |  |
| BDI | 19.9 | (12.3) |
| STAI-trait | 47.1 | (10.8) |
| Pathological DES | 19.0 | (24.4) |
| TAS | 56.7 | (13.3) |
| RSQ: Dismissing | 3.5 | (0.6) |
| RSQ: Fearful | 2.9 | (1.1) |
| RSQ: Preoccupied | 2.7 | (0.8) |
| RSQ: Secure | 3.0 | (0.6) |
| NEO: Agreeableness | 35.1 | (5.2) |
| NEO: Conscientiousness | 31.3 | (7.1) |
| NEO: Extraversion | 24.5 | (7.0) |
| NEO: Neuroticism | 26.6 | (8.9) |
| NEO: Openness | 26.5 | (5.6) |
| CDRS | 63.7 | (15.5) |

**Supplementary Table 1.**  In terms of missing data, one person did not complete their NEO or PCL-5; another also missed the PCL-5. BDI: Beck Depression Inventory I; DES: Dissociative Experiences Scale; CDRS: Connor-Davidson Resilience Scale; FND: Functional Neurological Disorder; FND-movt: Functional Movement Disorder; FND-seiz: Functional Seizures; NEO: NEO-Five Factor Inventory-3; RSQ: Relationship Scales Questionnaire; STAI-trait: Spielberger Trait Anxiety Inventory Subscale; SD: Standard Deviation; TAS: Toronto Alexithymia Scale; %: Percent of the total sample.

| **Linear Regressions to determine if FND-PTSD_high can predict Symptom Severity per SDQ-20 adjusting for demographic and other variables.** | | | | | | |
| --- | --- | --- | --- | --- | --- | --- |
| SDQ-20 | Coefficient | Std. Error | t | p-value | [95% Confidence Interval] | |
| Age | -0.141 | 0.078 | -1.798 | 0.076 | -0.297 | 0.015 |
| Female | -0.360 | 2.258 | -0.159 | 0.874 | -4.863 | 4.144 |
| White Race | -0.192 | 3.106 | -0.062 | 0.951 | -6.388 | 6.003 |
| College Graduate | -2.681 | 2.187 | -1.226 | 0.224 | -7.043 | 1.682 |
| FND-PTSD_high | 10.086 | 2.256 | 4.471 | **<0.001** | 5.586 | 14.585 |
|  |  |  |  |  |  |  |
| SDQ-20 | Coefficient | Std. Error | t | p-value | [95% Confidence Interval] | |
| Age | -0.141 | 0.077 | -1.821 | 0.073 | -0.296 | 0.014 |
| Female | -0.661 | 2.272 | -0.291 | 0.772 | -5.195 | 3.873 |
| White Race | 0.631 | 3.102 | 0.203 | 0.839 | -5.559 | 6.821 |
| College Graduate | -2.132 | 2.188 | -0.974 | 0.333 | -6.498 | 2.234 |
| Depression | 0.274 | 0.143 | 1.911 | 0.060 | -0.012 | 0.560 |
| Trait Anxiety | -0.135 | 0.155 | -0.870 | 0.387 | -0.444 | 0.174 |
| FND-PTSD_high | 7.818 | 2.677 | 2.921 | **0.005** | 2.477 | 13.160 |
|  |  |  |  |  |  |  |
| SDQ-20 | Coefficient | Std. Error | t | p-value | [95% Confidence Interval] | |
| Age | -0.017 | 0.070 | -0.250 | 0.804 | -0.157 | 0.122 |
| Female | 1.042 | 1.922 | 0.542 | 0.590 | -2.792 | 4.875 |
| White Race | 5.190 | 2.801 | 1.853 | 0.068 | -0.399 | 10.778 |
| College Graduate | -3.006 | 1.846 | -1.629 | 0.108 | -6.688 | 0.676 |
| Pathological Dissociation | 14.365 | 2.648 | 5.425 | <0.001 | 9.082 | 19.648 |
| FND-PTSD_high | 4.727 | 2.144 | 2.205 | **0.031** | 0.451 | 9.004 |
|  |  |  |  |  |  |  |
| SDQ-20 | Coefficient | Std. Error | t | p-value | [95% Confidence Interval] | |
| Age | -0.139 | 0.079 | -1.759 | 0.083 | -0.296 | 0.019 |
| Female | -0.238 | 2.294 | -0.104 | 0.918 | -4.814 | 4.338 |
| White Race | -0.040 | 3.150 | -0.013 | 0.990 | -6.325 | 6.244 |
| College Graduate | -2.480 | 2.262 | -1.096 | 0.277 | -6.992 | 2.032 |
| Alexithymia | 0.033 | 0.086 | 0.384 | 0.702 | -0.138 | 0.204 |
| FND-PTSD_high | 9.990 | 2.284 | 4.374 | **<0.001** | 5.434 | 14.545 |
|  |  |  |  |  |  |  |
| SDQ-20 | Coefficient | Std. Error | t | p-value | [95% Confidence Interval] | |
| Age | -0.149 | 0.075 | -1.989 | 0.051 | -0.299 | 0.001 |
| Female | -0.918 | 2.155 | -0.426 | 0.672 | -5.221 | 3.385 |
| White Race | 1.193 | 2.897 | 0.412 | 0.682 | -4.591 | 6.977 |
| College Graduate | -3.605 | 2.033 | -1.773 | 0.081 | -7.665 | 0.455 |
| RSQ-Dismissing Attachment Style | 0.242 | 2.039 | 0.119 | 0.906 | -3.828 | 4.313 |
| RSQ-Fearful Attachment Style | 3.120 | 1.070 | 2.917 | 0.005 | 0.984 | 5.257 |
| RSQ-Preoccupied Attachment Style | 1.240 | 1.356 | 0.914 | 0.364 | -1.467 | 3.947 |
| RSQ-Secure Attachment Style | 6.074 | 1.787 | 3.400 | 0.001 | 2.507 | 9.642 |
| FND-PTSD_high | 8.398 | 2.272 | 3.695 | **<0.001** | 3.861 | 12.935 |
|  |  |  |  |  |  |  |
|  |  |  |  |  |  |  |
|  |  |  |  |  |  |  |
| SDQ-20 | Coefficient | Std. Error | t | p-value | [95% Confidence Interval] | |
| Age | -0.168 | 0.082 | -2.041 | 0.045 | -0.332 | -0.004 |
| Female | -1.340 | 2.694 | -0.497 | 0.621 | -6.720 | 4.040 |
| White Race | 1.163 | 3.452 | 0.337 | 0.737 | -5.730 | 8.057 |
| College Graduate | -3.302 | 2.311 | -1.429 | 0.158 | -7.918 | 1.313 |
| NEO-Agreeableness Trait | 0.166 | 0.263 | 0.632 | 0.529 | -0.359 | 0.691 |
| NEO-Conscientiousness Trait | -0.156 | 0.184 | -0.850 | 0.399 | -0.524 | 0.211 |
| NEO-Extraversion Trait | 0.247 | 0.172 | 1.435 | 0.156 | -0.097 | 0.590 |
| NEO-Neuroticism Trait | 0.048 | 0.150 | 0.319 | 0.751 | -0.252 | 0.348 |
| NEO-Openness Trait | 0.286 | 0.210 | 1.362 | 0.178 | -0.133 | 0.706 |
| FND-PTSD_high | 10.513 | 2.568 | 4.093 | **<0.001** | 5.384 | 15.641 |
|  |  |  |  |  |  |  |
| SDQ-20 | Coefficient | Std. Error | t | p-value | [95% Confidence Interval] | |
| Age | -0.139 | 0.077 | -1.809 | 0.075 | -0.293 | 0.014 |
| Female | -0.118 | 2.227 | -0.053 | 0.958 | -4.562 | 4.325 |
| White Race | -0.105 | 3.059 | -0.034 | 0.973 | -6.207 | 5.996 |
| College Graduate | -4.091 | 2.292 | -1.784 | 0.079 | -8.664 | 0.483 |
| CDRS-Resilience | 0.135 | 0.075 | 1.793 | 0.077 | -0.015 | 0.285 |
| FND-PTSD_high | 11.251 | 2.314 | 4.862 | **<0.001** | 6.634 | 15.868 |
|  |  |  |  |  |  |  |
| SDQ-20 | Coefficient | Std. Error | t | p-value | [95% Confidence Interval] | |
| Age | -0.144 | 0.083 | -1.746 | 0.085 | -0.309 | 0.021 |
| Female | -0.342 | 2.277 | -0.150 | 0.881 | -4.886 | 4.201 |
| White Race | -0.272 | 3.179 | -0.086 | 0.932 | -6.614 | 6.070 |
| College Graduate | -2.698 | 2.206 | -1.223 | 0.226 | -7.099 | 1.703 |
| FND-seiz | -0.322 | 2.279 | -0.141 | 0.888 | -4.869 | 4.224 |
| FND-PTSD_high | 10.080 | 2.272 | 4.436 | **<0.001** | 5.547 | 14.613 |
|  |  |  |  |  |  |  |
| SDQ-20 | Coefficient | Std. Error | t | p-value | [95% Confidence Interval] | |
| Age | -0.170 | 0.077 | -2.214 | 0.030 | -0.324 | -0.017 |
| Female | -0.572 | 2.202 | -0.260 | 0.796 | -4.966 | 3.822 |
| White Race | 0.460 | 3.058 | 0.150 | 0.881 | -5.641 | 6.561 |
| College Graduate | -2.665 | 2.131 | -1.250 | 0.215 | -6.918 | 1.588 |
| FND-Abuse_high | 5.228 | 2.551 | 2.050 | 0.044 | 0.138 | 10.318 |
| FND-Neglect_high | 0.107 | 2.465 | 0.044 | 0.965 | -4.811 | 5.025 |
| FND-PTSD_high | 9.004 | 2.236 | 4.026 | **<0.001** | 4.541 | 13.466 |

**Supplementary Table 2.** Linear regressions to test if FND-PTSD_high can predict symptom severity per SDQ-20 adjusting for demographic and other neuropsychiatric variables. P-values less than or equal to 0.05 are in bold and represent statistical significance. FND: Functional Neurological Disorder; FND-Abuse_high: subgroup of participants with FND with Childhood Trauma Questionnaire subscale scores for abuse at or above cut-off; FND-Neglect_high: subgroup of participants with FND with Childhood Trauma Questionnaire subscale scores for neglect at or above cut-off; FND-PTSD_high: subgroup of participants with FND with PTSD symptoms per the PTSD Checklist for DSM-5 indicative of possible PTSD; SDQ-20: Somatoform Dissociation Questionnaire, a measure of symptom severity; Std. Error: Standard Error; t: t-statistics for linear regression.

| **Linear Regressions to determine if FND-PTSD_high can predict Symptom Severity per PHQ-15 adjusting for demographic and other variables.** | | | | | | |
| --- | --- | --- | --- | --- | --- | --- |
| PHQ-15 | Coefficient | Std. Error | t | p-value | [95% Confidence Interval] | |
| Age | -0.068 | 0.038 | -1.795 | 0.077 | -0.144 | 0.008 |
| Female | 0.826 | 1.097 | 0.753 | 0.454 | -1.362 | 3.013 |
| White Race | 0.362 | 1.509 | 0.240 | 0.811 | -2.648 | 3.371 |
| College Graduate | 0.181 | 1.063 | 0.170 | 0.865 | -1.939 | 2.300 |
| FND-PTSD_high | 4.555 | 1.096 | 4.156 | **<0.001** | 2.369 | 6.741 |
|  |  |  |  |  |  |  |
| PHQ-15 | Coefficient | Std. Error | t | p-value | [95% Confidence Interval] | |
| Age | -0.067 | 0.035 | -1.915 | 0.060 | -0.136 | 0.003 |
| Female | 0.428 | 1.018 | 0.420 | 0.676 | -1.604 | 2.460 |
| White Race | 1.180 | 1.390 | 0.848 | 0.399 | -1.595 | 3.954 |
| College Graduate | 0.747 | 0.981 | 0.762 | 0.449 | -1.210 | 2.704 |
| Depression | 0.238 | 0.064 | 3.714 | <0.001 | 0.110 | 0.367 |
| Trait Anxiety | -0.082 | 0.069 | -1.176 | 0.244 | -0.220 | 0.057 |
| FND-PTSD_high | 2.215 | 1.200 | 1.847 | 0.069 | -0.179 | 4.609 |
|  |  |  |  |  |  |  |
| PHQ-15 | Coefficient | Std. Error | t | p-value | [95% Confidence Interval] | |
| Age | -0.036 | 0.039 | -0.920 | 0.361 | -0.113 | 0.042 |
| Female | 1.197 | 1.064 | 1.125 | 0.265 | -0.926 | 3.320 |
| White Race | 1.788 | 1.551 | 1.153 | 0.253 | -1.307 | 4.883 |
| College Graduate | 0.094 | 1.022 | 0.092 | 0.927 | -1.945 | 2.133 |
| Pathological Dissociation | 3.808 | 1.467 | 2.596 | 0.012 | 0.882 | 6.734 |
| FND-PTSD_high | 3.135 | 1.187 | 2.640 | **0.010** | 0.766 | 5.503 |
|  |  |  |  |  |  |  |
| PHQ-15 | Coefficient | Std. Error | t | p-value | [95% Confidence Interval] | |
| Age | -0.067 | 0.038 | -1.743 | 0.086 | -0.143 | 0.010 |
| Female | 0.922 | 1.112 | 0.829 | 0.410 | -1.297 | 3.141 |
| White Race | 0.482 | 1.528 | 0.315 | 0.753 | -2.566 | 3.530 |
| College Graduate | 0.339 | 1.097 | 0.309 | 0.758 | -1.849 | 2.528 |
| Alexithymia | 0.026 | 0.042 | 0.627 | 0.533 | -0.057 | 0.109 |
| FND-PTSD_high | 4.479 | 1.108 | 4.044 | **<0.001** | 2.269 | 6.688 |
|  |  |  |  |  |  |  |
| PHQ-15 | Coefficient | Std. Error | t | p-value | [95% Confidence Interval] | |
| Age | -0.058 | 0.038 | -1.535 | 0.130 | -0.133 | 0.017 |
| Female | 0.679 | 1.080 | 0.629 | 0.532 | -1.478 | 2.836 |
| White Race | 0.899 | 1.452 | 0.619 | 0.538 | -2.000 | 3.798 |
| College Graduate | -0.070 | 1.019 | -0.069 | 0.945 | -2.105 | 1.965 |
| RSQ-Dismissing Attachment Style | -0.900 | 1.022 | -0.880 | 0.382 | -2.940 | 1.141 |
| RSQ-Fearful Attachment Style | 1.417 | 0.536 | 2.642 | 0.010 | 0.346 | 2.488 |
| RSQ-Preoccupied Attachment Style | 0.986 | 0.680 | 1.451 | 0.152 | -0.371 | 2.343 |
| RSQ-Secure Attachment Style | 2.052 | 0.896 | 2.291 | 0.025 | 0.263 | 3.840 |
| FND-PTSD_high | 3.693 | 1.139 | 3.242 | **0.002** | 1.419 | 5.968 |
|  |  |  |  |  |  |  |
| PHQ-15 | Coefficient | Std. Error | t | p-value | [95% Confidence Interval] | |
| Age | -0.085 | 0.040 | -2.100 | 0.040 | -0.166 | -0.004 |
| Female | -0.135 | 1.326 | -0.102 | 0.919 | -2.782 | 2.513 |
| White Race | 1.027 | 1.699 | 0.604 | 0.548 | -2.366 | 4.419 |
| College Graduate | 0.103 | 1.137 | 0.090 | 0.928 | -2.169 | 2.375 |
| NEO-Agreeableness Trait | 0.131 | 0.129 | 1.016 | 0.313 | -0.127 | 0.390 |
| NEO-Conscientiousness Trait | -0.018 | 0.091 | -0.201 | 0.841 | -0.199 | 0.163 |
| NEO-Extraversion Trait | -0.054 | 0.085 | -0.638 | 0.526 | -0.223 | 0.115 |
| NEO-Neuroticism Trait | 0.030 | 0.074 | 0.411 | 0.682 | -0.117 | 0.178 |
| NEO-Openness Trait | 0.086 | 0.103 | 0.830 | 0.410 | -0.121 | 0.292 |
| FND-PTSD_high | 4.008 | 1.264 | 3.171 | **0.002** | 1.484 | 6.533 |
|  |  |  |  |  |  |  |
| PHQ-15 | Coefficient | Std. Error | t | p-value | [95% Confidence Interval] | |
| Age | -0.068 | 0.038 | -1.782 | 0.079 | -0.144 | 0.008 |
| Female | 0.873 | 1.103 | 0.791 | 0.431 | -1.328 | 3.073 |
| White Race | 0.379 | 1.515 | 0.250 | 0.803 | -2.643 | 3.400 |
| College Graduate | -0.095 | 1.135 | -0.084 | 0.933 | -2.360 | 2.169 |
| CDRS-Resilience | 0.026 | 0.037 | 0.709 | 0.481 | -0.048 | 0.101 |
| FND-PTSD_high | 4.783 | 1.146 | 4.174 | **<0.001** | 2.497 | 7.070 |
|  |  |  |  |  |  |  |
| PHQ-15 | Coefficient | Std. Error | t | p-value | [95% Confidence Interval] | |
| Age | -0.075 | 0.040 | -1.865 | 0.066 | -0.154 | 0.005 |
| Female | 0.858 | 1.104 | 0.777 | 0.440 | -1.345 | 3.061 |
| White Race | 0.214 | 1.541 | 0.139 | 0.890 | -2.861 | 3.289 |
| College Graduate | 0.149 | 1.070 | 0.139 | 0.890 | -1.985 | 2.283 |
| FND-seiz | -0.595 | 1.105 | -0.539 | 0.592 | -2.800 | 1.609 |
| FND-PTSD_high | 4.545 | 1.102 | 4.125 | **<0.001** | 2.347 | 6.743 |
|  |  |  |  |  |  |  |
| PHQ-15 | Coefficient | Std. Error | t | p-value | [95% Confidence Interval] | |
| Age | -0.087 | 0.036 | -2.422 | 0.018 | -0.159 | -0.015 |
| Female | 0.583 | 1.030 | 0.565 | 0.574 | -1.473 | 2.639 |
| White Race | 1.044 | 1.431 | 0.730 | 0.468 | -1.811 | 3.899 |
| College Graduate | 0.306 | 0.997 | 0.307 | 0.760 | -1.684 | 2.297 |
| FND-Abuse_high | 4.091 | 1.194 | 3.427 | 0.001 | 1.709 | 6.473 |
| FND-Neglect_high | -1.792 | 1.153 | -1.554 | 0.125 | -4.093 | 0.509 |
| FND-PTSD_high | 3.967 | 1.047 | 3.791 | **<0.001** | 1.879 | 6.055 |

**Supplementary Table 3.** Linear regressions to test if FND-PTSD_high can predict symptom severity per PHQ-15 adjusting for demographic and other neuropsychiatric. P-values less than or equal to 0.05 are in bold and represent statistical significance. FND: Functional Neurological Disorder; FND-Abuse_high: subgroup of participants with FND with Childhood Trauma Questionnaire subscale scores for abuse at or above cut-off; FND-Neglect_high: subgroup of participants with FND with Childhood Trauma Questionnaire subscale scores for neglect at or above cut-off; FND-PTSD_high: subgroup of participants with FND with PTSD symptoms per the PTSD Checklist for DSM-5 indicative of possible PTSD; PHQ-15: Patient Health Questionnaire-15, a measure of symptom severity; Std. Error: Standard Error; t: t-statistics for linear regression.

| **Linear Regressions to determine if FND-PTSD_high can predict Symptom Severity per SOMS:CD adjusting for demographic and other variables.** | | | | | | |
| --- | --- | --- | --- | --- | --- | --- |
| SOMS:CD | Coefficient | Std. Error | t | p-value | [95% Confidence Interval] | |
| Age | -0.048 | 0.054 | -0.887 | 0.378 | -0.155 | 0.060 |
| Female | -0.661 | 1.559 | -0.424 | 0.673 | -3.770 | 2.447 |
| White Race | -1.495 | 2.144 | -0.698 | 0.488 | -5.771 | 2.781 |
| College Graduate | -1.809 | 1.510 | -1.198 | 0.235 | -4.820 | 1.202 |
| FND-PTSD_high | 5.495 | 1.557 | 3.529 | **0.001** | 2.390 | 8.601 |
|  |  |  |  |  |  |  |
| SOMS:CD | Coefficient | Std. Error | t | p-value | [95% Confidence Interval] | |
| Age | -0.053 | 0.054 | -0.975 | 0.333 | -0.160 | 0.055 |
| Female | -0.541 | 1.582 | -0.342 | 0.733 | -3.697 | 2.615 |
| White Race | -1.247 | 2.159 | -0.577 | 0.566 | -5.555 | 3.062 |
| College Graduate | -1.689 | 1.523 | -1.109 | 0.271 | -4.728 | 1.350 |
| Depression | 0.155 | 0.100 | 1.553 | 0.125 | -0.044 | 0.354 |
| Trait Anxiety | -0.153 | 0.108 | -1.419 | 0.160 | -0.368 | 0.062 |
| FND-PTSD_high | 4.998 | 1.863 | 2.683 | **0.009** | 1.281 | 8.716 |
|  |  |  |  |  |  |  |
| SOMS:CD | Coefficient | Std. Error | t | p-value | [95% Confidence Interval] | |
| Age | -0.001 | 0.055 | -0.025 | 0.980 | -0.111 | 0.108 |
| Female | -0.133 | 1.512 | -0.088 | 0.930 | -3.149 | 2.883 |
| White Race | 0.535 | 2.204 | 0.243 | 0.809 | -3.862 | 4.931 |
| College Graduate | -1.931 | 1.452 | -1.330 | 0.188 | -4.828 | 0.965 |
| Pathological Dissociation | 5.418 | 2.083 | 2.601 | 0.011 | 1.262 | 9.574 |
| FND-PTSD_high | 3.474 | 1.686 | 2.060 | **0.043** | 0.110 | 6.839 |
|  |  |  |  |  |  |  |
| SOMS:CD | Coefficient | Std. Error | t | p-value | [95% Confidence Interval] | |
| Age | -0.052 | 0.054 | -0.963 | 0.339 | -0.160 | 0.056 |
| Female | -0.913 | 1.570 | -0.582 | 0.563 | -4.045 | 2.218 |
| White Race | -1.810 | 2.156 | -0.840 | 0.404 | -6.111 | 2.490 |
| College Graduate | -2.224 | 1.548 | -1.437 | 0.155 | -5.312 | 0.864 |
| Alexithymia | -0.068 | 0.059 | -1.162 | 0.249 | -0.186 | 0.049 |
| FND-PTSD_high | 5.695 | 1.563 | 3.644 | **0.001** | 2.577 | 8.812 |
|  |  |  |  |  |  |  |
| SOMS:CD | Coefficient | Std. Error | t | p-value | [95% Confidence Interval] | |
| Age | -0.079 | 0.054 | -1.456 | 0.150 | -0.187 | 0.029 |
| Female | -1.000 | 1.555 | -0.643 | 0.522 | -4.105 | 2.104 |
| White Race | -0.407 | 2.090 | -0.195 | 0.846 | -4.580 | 3.765 |
| College Graduate | -2.665 | 1.467 | -1.817 | 0.074 | -5.594 | 0.264 |
| RSQ-Dismissing Attachment Style | 2.954 | 1.471 | 2.008 | 0.049 | 0.017 | 5.890 |
| RSQ-Fearful Attachment Style | -0.136 | 0.772 | -0.176 | 0.861 | -1.677 | 1.405 |
| RSQ-Preoccupied Attachment Style | 1.035 | 0.978 | 1.058 | 0.294 | -0.918 | 2.988 |
| RSQ-Secure Attachment Style | 3.747 | 1.289 | 2.907 | 0.005 | 1.174 | 6.321 |
| FND-PTSD_high | 6.158 | 1.639 | 3.756 | **<0.001** | 2.884 | 9.431 |
| SOMS:CD | Coefficient | Std. Error | t | p-value | [95% Confidence Interval] | |
| Age | -0.045 | 0.056 | -0.792 | 0.431 | -0.157 | 0.068 |
| Female | -0.466 | 1.843 | -0.253 | 0.801 | -4.147 | 3.214 |
| White Race | -2.127 | 2.361 | -0.901 | 0.371 | -6.843 | 2.589 |
| College Graduate | -1.342 | 1.581 | -0.849 | 0.399 | -4.500 | 1.815 |
| NEO-Agreeableness Trait | 0.096 | 0.180 | 0.532 | 0.596 | -0.263 | 0.455 |
| NEO-Conscientiousness Trait | -0.168 | 0.126 | -1.337 | 0.186 | -0.419 | 0.083 |
| NEO-Extraversion Trait | 0.220 | 0.118 | 1.873 | 0.066 | -0.015 | 0.455 |
| NEO-Neuroticism Trait | -0.061 | 0.103 | -0.590 | 0.557 | -0.266 | 0.145 |
| NEO-Openness Trait | -0.093 | 0.144 | -0.644 | 0.522 | -0.380 | 0.194 |
| FND-PTSD_high | 6.946 | 1.757 | 3.953 | **<0.001** | 3.437 | 10.455 |
|  |  |  |  |  |  |  |
| SOMS:CD | Coefficient | Std. Error | t | p-value | [95% Confidence Interval] | |
| Age | -0.047 | 0.054 | -0.877 | 0.383 | -0.155 | 0.060 |
| Female | -0.560 | 1.560 | -0.359 | 0.721 | -3.671 | 2.552 |
| White Race | -1.459 | 2.142 | -0.681 | 0.498 | -5.732 | 2.814 |
| College Graduate | -2.402 | 1.605 | -1.496 | 0.139 | -5.605 | 0.800 |
| CDRS-Resilience | 0.057 | 0.053 | 1.078 | 0.285 | -0.048 | 0.162 |
| FND-PTSD_high | 5.986 | 1.621 | 3.694 | **<0.001** | 2.753 | 9.218 |
|  |  |  |  |  |  |  |
| SOMS:CD | Coefficient | Std. Error | t | p-value | [95% Confidence Interval] | |
| Age | -0.058 | 0.057 | -1.023 | 0.310 | -0.172 | 0.055 |
| Female | -0.610 | 1.568 | -0.389 | 0.698 | -3.738 | 2.518 |
| White Race | -1.731 | 2.189 | -0.791 | 0.432 | -6.097 | 2.636 |
| College Graduate | -1.859 | 1.519 | -1.224 | 0.225 | -4.889 | 1.171 |
| FND-seiz | -0.948 | 1.569 | -0.604 | 0.548 | -4.078 | 2.183 |
| FND-PTSD_high | 5.479 | 1.565 | 3.502 | **0.001** | 2.358 | 8.600 |
|  |  |  |  |  |  |  |
| SOMS:CD | Coefficient | Std. Error | t | p-value | [95% Confidence Interval] | |
| Age | -0.059 | 0.054 | -1.089 | 0.280 | -0.166 | 0.049 |
| Female | -0.918 | 1.541 | -0.595 | 0.553 | -3.993 | 2.158 |
| White Race | -0.814 | 2.140 | -0.381 | 0.705 | -5.085 | 3.456 |
| College Graduate | -1.610 | 1.492 | -1.079 | 0.284 | -4.586 | 1.367 |
| FND-Abuse_high | 3.157 | 1.785 | 1.769 | 0.081 | -0.405 | 6.720 |
| FND-Neglect_high | -3.068 | 1.725 | -1.779 | 0.080 | -6.510 | 0.374 |
| FND-PTSD_high | 5.274 | 1.565 | 3.369 | **0.001** | 2.150 | 8.397 |

**Supplementary Table 4.** Linear regressions to test if FND-PTSD_high can predict symptom severity per SOMS:CD adjusting for demographic and other neuropsychiatric variables. P-values less than or equal to 0.05 are in bold and represent statistical significance. FND: Functional Neurological Disorder; FND-Abuse_high: subgroup of participants with FND with Childhood Trauma Questionnaire subscale scores for abuse at or above cut-off; FND-Neglect_high: subgroup of participants with FND with Childhood Trauma Questionnaire subscale scores for neglect at or above cut-off; FND-PTSD_high: subgroup of participants with FND with PTSD symptoms per the PTSD Checklist for DSM-5 indicative of possible PTSD; SOMS:CD: Somatoform Symptoms-7 subscale for Conversion Disorder, a measure of symptom severity; Std. Error: Standard Error; t: t-statistics for linear regression.

| **Linear Regressions to determine if FND-PTSD_high can predict Physical Health per the SF36 Physical Health Component Score adjusting for demographic and other variables.** | | | | | | |
| --- | --- | --- | --- | --- | --- | --- |
|  |  |  |  |  |  |  |
| SF36 Physical Health | Coefficient | Std. Error | t | p-value | [95% Confidence Interval] | |
| Age | -0.109 | 0.184 | -0.589 | 0.558 | -0.476 | 0.259 |
| Female | -3.404 | 5.326 | -0.639 | 0.525 | -14.025 | 7.218 |
| White Race | -7.015 | 7.326 | -0.958 | 0.342 | -21.626 | 7.596 |
| College Graduate | 2.524 | 5.159 | 0.489 | 0.626 | -7.765 | 12.812 |
| FND-PTSD_high | -15.675 | 5.321 | -2.946 | **0.004** | -26.287 | -5.063 |
|  |  |  |  |  |  |  |
| SF36 Physical Health | Coefficient | Std. Error | t | p-value | [95% Confidence Interval] | |
| Age | -0.104 | 0.182 | -0.572 | 0.569 | -0.468 | 0.260 |
| Female | -2.864 | 5.350 | -0.535 | 0.594 | -13.540 | 7.811 |
| White Race | -8.901 | 7.303 | -1.219 | 0.227 | -23.475 | 5.673 |
| College Graduate | 1.300 | 5.151 | 0.252 | 0.802 | -8.979 | 11.579 |
| Depression | -0.679 | 0.337 | -2.012 | 0.048 | -1.352 | -0.005 |
| Trait Anxiety | 0.389 | 0.365 | 1.066 | 0.290 | -0.339 | 1.116 |
| FND-PTSD_high | -10.616 | 6.302 | -1.685 | 0.097 | -23.191 | 1.959 |
|  |  |  |  |  |  |  |
| SF36 Physical Health | Coefficient | Std. Error | t | p-value | [95% Confidence Interval] | |
| Age | -0.181 | 0.195 | -0.929 | 0.356 | -0.569 | 0.208 |
| Female | -4.224 | 5.363 | -0.788 | 0.434 | -14.923 | 6.475 |
| White Race | -10.164 | 7.818 | -1.300 | 0.198 | -25.760 | 5.431 |
| College Graduate | 2.714 | 5.151 | 0.527 | 0.600 | -7.561 | 12.989 |
| Pathological Dissociation | -8.406 | 7.390 | -1.137 | 0.259 | -23.150 | 6.338 |
| FND-PTSD_high | -12.540 | 5.983 | -2.096 | **0.040** | -24.475 | -0.604 |
|  |  |  |  |  |  |  |
| SF36 Physical Health | Coefficient | Std. Error | t | p-value | [95% Confidence Interval] | |
| Age | -0.097 | 0.185 | -0.525 | 0.601 | -0.466 | 0.272 |
| Female | -2.687 | 5.380 | -0.500 | 0.619 | -13.420 | 8.045 |
| White Race | -6.120 | 7.388 | -0.828 | 0.410 | -20.859 | 8.619 |
| College Graduate | 3.704 | 5.305 | 0.698 | 0.487 | -6.878 | 14.286 |
| Alexithymia | 0.194 | 0.201 | 0.964 | 0.338 | -0.208 | 0.596 |
| FND-PTSD_high | -16.243 | 5.356 | -3.033 | **0.003** | -26.927 | -5.558 |
|  |  |  |  |  |  |  |
| SF36 Physical Health | Coefficient | Std. Error | t | p-value | [95% Confidence Interval] | |
| Age | -0.075 | 0.192 | -0.390 | 0.697 | -0.459 | 0.309 |
| Female | -3.550 | 5.518 | -0.643 | 0.522 | -14.568 | 7.467 |
| White Race | -9.295 | 7.417 | -1.253 | 0.215 | -24.104 | 5.514 |
| College Graduate | 4.148 | 5.206 | 0.797 | 0.428 | -6.247 | 14.543 |
| RSQ-Dismissing Attachment Style | -1.857 | 5.220 | -0.356 | 0.723 | -12.279 | 8.565 |
| RSQ-Fearful Attachment Style | -1.194 | 2.740 | -0.436 | 0.664 | -6.664 | 4.276 |
| RSQ-Preoccupied Attachment Style | -3.453 | 3.472 | -0.995 | 0.324 | -10.385 | 3.478 |
| RSQ-Secure Attachment Style | -9.976 | 4.575 | -2.180 | 0.033 | -19.110 | -0.841 |
| FND-PTSD_high | -16.400 | 5.818 | -2.819 | **0.006** | -28.017 | -4.783 |
|  |  |  |  |  |  |  |
| SF36 Physical Health | Coefficient | Std. Error | t | p-value | [95% Confidence Interval] | |
| Age | -0.094 | 0.198 | -0.472 | 0.639 | -0.490 | 0.303 |
| Female | -2.919 | 6.508 | -0.449 | 0.655 | -15.917 | 10.078 |
| White Race | -6.285 | 8.339 | -0.754 | 0.454 | -22.940 | 10.370 |
| College Graduate | 2.308 | 5.584 | 0.413 | 0.681 | -8.844 | 13.460 |
| NEO-Agreeableness Trait | -0.246 | 0.635 | -0.387 | 0.700 | -1.514 | 1.022 |
| NEO-Conscientiousness Trait | 0.261 | 0.444 | 0.587 | 0.559 | -0.627 | 1.148 |
| NEO-Extraversion Trait | -0.434 | 0.415 | -1.046 | 0.299 | -1.263 | 0.395 |
| NEO-Neuroticism Trait | 0.114 | 0.363 | 0.315 | 0.754 | -0.610 | 0.839 |
| NEO-Openness Trait | -0.035 | 0.508 | -0.068 | 0.946 | -1.048 | 0.979 |
| FND-PTSD_high | -18.307 | 6.205 | -2.950 | **0.004** | -30.699 | -5.915 |
|  |  |  |  |  |  |  |
| SF36 Physical Health | Coefficient | Std. Error | t | p-value | [95% Confidence Interval] | |
| Age | -0.110 | 0.185 | -0.596 | 0.553 | -0.479 | 0.258 |
| Female | -3.681 | 5.345 | -0.689 | 0.493 | -14.345 | 6.983 |
| White Race | -7.115 | 7.341 | -0.969 | 0.336 | -21.760 | 7.530 |
| College Graduate | 4.141 | 5.502 | 0.753 | 0.454 | -6.835 | 15.118 |
| CDRS-Resilience | -0.155 | 0.181 | -0.857 | 0.394 | -0.515 | 0.206 |
| FND-PTSD_high | -17.012 | 5.554 | -3.063 | **0.003** | -28.092 | -5.932 |
|  |  |  |  |  |  |  |
| SF36 Physical Health | Coefficient | Std. Error | t | p-value | [95% Confidence Interval] | |
| Age | -0.005 | 0.190 | -0.027 | 0.979 | -0.385 | 0.374 |
| Female | -3.919 | 5.248 | -0.747 | 0.458 | -14.389 | 6.551 |
| White Race | -4.648 | 7.326 | -0.634 | 0.528 | -19.263 | 9.967 |
| College Graduate | 3.032 | 5.084 | 0.596 | 0.553 | -7.110 | 13.174 |
| FND-seiz | 9.528 | 5.252 | 1.814 | 0.074 | -0.949 | 20.005 |
| FND-PTSD_high | -15.508 | 5.237 | -2.962 | **0.004** | -25.955 | -5.062 |
|  |  |  |  |  |  |  |
| SF36 Physical Health | Coefficient | Std. Error | t | p-value | [95% Confidence Interval] | |
| Age | -0.066 | 0.186 | -0.354 | 0.725 | -0.437 | 0.306 |
| Female | -2.791 | 5.327 | -0.524 | 0.602 | -13.420 | 7.838 |
| White Race | -8.715 | 7.396 | -1.178 | 0.243 | -23.473 | 6.044 |
| College Graduate | 2.168 | 5.156 | 0.421 | 0.675 | -8.120 | 12.456 |
| FND-Abuse_high | -9.665 | 6.170 | -1.566 | 0.122 | -21.977 | 2.648 |
| FND-Neglect_high | 5.194 | 5.961 | 0.871 | 0.387 | -6.701 | 17.090 |
| FND-PTSD_high | -14.418 | 5.410 | -2.665 | **0.010** | -25.213 | -3.623 |

**Supplementary Table 5**. Linear regressions to test if FND-PTSD_high can predict physical health component score per SF36 adjusting for demographic and other neuropsychiatric variables. P-values less than or equal to 0.05 are in bold and represent statistical significance. FND: Functional Neurological Disorder; FND-Abuse_high: subgroup of participants with FND with Childhood Trauma Questionnaire subscale scores for abuse at or above cut-off; FND-Neglect_high: subgroup of participants with FND with Childhood Trauma Questionnaire subscale scores for neglect at or above cut-off; FND-PTSD_high: subgroup of participants with FND with PTSD symptoms per the PTSD Checklist for DSM-5 indicative of possible PTSD; SF36: Short Form Health Survey-36, a measure of physical health; Std. Error: Standard Error; t: t-statistics for linear regression.

| **Linear Regressions to determine if FND-Abuse_high can predict Symptom Severity per SDQ-20 adjusting for demographic and other variables.** | | | | | | |
| --- | --- | --- | --- | --- | --- | --- |
|  |  |  |  |  |  |  |
| SDQ-20 | Coefficient | Std. Error | t | p-value | [95% Confidence Interval] | |
| Age | -0.142 | 0.082 | -1.734 | 0.087 | -0.305 | 0.021 |
| Female | -0.820 | 2.342 | -0.350 | 0.727 | -5.488 | 3.848 |
| White Race | -2.487 | 3.190 | -0.780 | 0.438 | -8.847 | 3.872 |
| College Graduate | -4.762 | 2.190 | -2.174 | 0.033 | -9.128 | -0.396 |
| FND-Abuse_high | 7.179 | 2.241 | 3.203 | **0.002** | 2.711 | 11.647 |
|  |  |  |  |  |  |  |
| SDQ-20 | Coefficient | Std. Error | t | p-value | [95% Confidence Interval] | |
| Age | -0.137 | 0.078 | -1.761 | 0.083 | -0.293 | 0.018 |
| Female | -1.445 | 2.243 | -0.644 | 0.522 | -5.918 | 3.029 |
| White Race | -0.200 | 3.122 | -0.064 | 0.949 | -6.426 | 6.026 |
| College Graduate | -2.977 | 2.161 | -1.378 | 0.173 | -7.286 | 1.332 |
| Depression | 0.359 | 0.136 | 2.637 | 0.010 | 0.087 | 0.630 |
| Trait Anxiety | -0.098 | 0.152 | -0.648 | 0.519 | -0.401 | 0.204 |
| FND-Abuse_high | 5.022 | 2.286 | 2.196 | **0.031** | 0.462 | 9.582 |
|  |  |  |  |  |  |  |
| SDQ-20 | Coefficient | Std. Error | t | p-value | [95% Confidence Interval] | |
| Age | -0.015 | 0.068 | -0.216 | 0.829 | -0.151 | 0.121 |
| Female | 0.781 | 1.882 | 0.415 | 0.679 | -2.971 | 4.534 |
| White Race | 4.935 | 2.786 | 1.771 | 0.081 | -0.620 | 10.490 |
| College Graduate | -3.692 | 1.753 | -2.107 | 0.039 | -7.187 | -0.198 |
| Pathological Dissociation | 15.751 | 2.419 | 6.511 | <0.001 | 10.928 | 20.575 |
| FND-Abuse_high | 4.110 | 1.847 | 2.225 | **0.029** | 0.427 | 7.793 |
|  |  |  |  |  |  |  |
| SDQ-20 | Coefficient | Std. Error | t | p-value | [95% Confidence Interval] | |
| Age | -0.140 | 0.083 | -1.689 | 0.096 | -0.304 | 0.025 |
| Female | -0.623 | 2.397 | -0.260 | 0.796 | -5.402 | 4.156 |
| White Race | -2.299 | 3.236 | -0.710 | 0.480 | -8.752 | 4.154 |
| College Graduate | -4.545 | 2.256 | -2.015 | 0.048 | -9.044 | -0.046 |
| Alexithymia | 0.039 | 0.088 | 0.442 | 0.660 | -0.137 | 0.215 |
| FND-Abuse_high | 6.998 | 2.291 | 3.055 | **0.003** | 2.430 | 11.566 |
|  |  |  |  |  |  |  |
| SDQ-20 | Coefficient | Std. Error | t | p-value | [95% Confidence Interval] | |
| Age | -0.148 | 0.079 | -1.869 | 0.066 | -0.305 | 0.010 |
| Female | -1.751 | 2.251 | -0.778 | 0.439 | -6.242 | 2.740 |
| White Race | -0.902 | 2.996 | -0.301 | 0.764 | -6.881 | 5.077 |
| College Graduate | -4.734 | 2.065 | -2.293 | 0.025 | -8.853 | -0.614 |
| RSQ-Dismissing Attachment Style | -0.144 | 2.124 | -0.068 | 0.946 | -4.383 | 4.095 |
| RSQ-Fearful Attachment Style | 3.717 | 1.098 | 3.384 | 0.001 | 1.525 | 5.909 |
| RSQ-Preoccupied Attachment Style | 0.440 | 1.424 | 0.309 | 0.758 | -2.401 | 3.281 |
| RSQ-Secure Attachment Style | 5.335 | 1.857 | 2.873 | 0.005 | 1.630 | 9.040 |
| FND-Abuse_high | 5.488 | 2.258 | 2.430 | **0.018** | 0.982 | 9.993 |
|  |  |  |  |  |  |  |
| SDQ-20 | Coefficient | Std. Error | t | p-value | [95% Confidence Interval] | |
| Age | -0.151 | 0.087 | -1.731 | 0.088 | -0.326 | 0.023 |
| Female | -1.145 | 2.894 | -0.396 | 0.694 | -6.923 | 4.633 |
| White Race | -0.108 | 3.680 | -0.029 | 0.977 | -7.456 | 7.239 |
| College Graduate | -5.343 | 2.367 | -2.257 | 0.027 | -10.069 | -0.617 |
| NEO-Agreeableness Trait | -0.034 | 0.285 | -0.120 | 0.905 | -0.603 | 0.535 |
| NEO-Conscientiousness Trait | -0.012 | 0.195 | -0.063 | 0.950 | -0.401 | 0.376 |
| NEO-Extraversion Trait | 0.056 | 0.181 | 0.312 | 0.756 | -0.304 | 0.417 |
| NEO-Neuroticism Trait | 0.196 | 0.153 | 1.284 | 0.204 | -0.109 | 0.502 |
| NEO-Openness Trait | 0.254 | 0.227 | 1.118 | 0.268 | -0.199 | 0.707 |
| FND-Abuse_high | 5.970 | 2.434 | 2.452 | **0.017** | 1.110 | 10.830 |
|  |  |  |  |  |  |  |
| SDQ-20 | Coefficient | Std. Error | t | p-value | [95% Confidence Interval] | |
| Age | -0.141 | 0.082 | -1.708 | 0.092 | -0.305 | 0.024 |
| Female | -0.795 | 2.353 | -0.338 | 0.736 | -5.487 | 3.896 |
| White Race | -2.574 | 3.208 | -0.802 | 0.425 | -8.972 | 3.823 |
| College Graduate | -5.280 | 2.370 | -2.228 | 0.029 | -10.006 | -0.554 |
| CDRS-Resilience | 0.045 | 0.076 | 0.588 | 0.559 | -0.108 | 0.197 |
| FND-Abuse_high | 7.341 | 2.268 | 3.236 | **0.002** | 2.818 | 11.864 |
|  |  |  |  |  |  |  |
| SDQ-20 | Coefficient | Std. Error | t | p-value | [95% Confidence Interval] | |
| Age | -0.148 | 0.087 | -1.701 | 0.093 | -0.321 | 0.026 |
| Female | -0.794 | 2.361 | -0.336 | 0.738 | -5.501 | 3.913 |
| White Race | -2.610 | 3.263 | -0.800 | 0.426 | -9.116 | 3.897 |
| College Graduate | -4.784 | 2.207 | -2.168 | 0.034 | -9.185 | -0.383 |
| FND-seiz | -0.508 | 2.392 | -0.212 | 0.833 | -5.278 | 4.262 |
| FND-Abuse_high | 7.193 | 2.257 | 3.186 | **0.002** | 2.692 | 11.694 |
|  |  |  |  |  |  |  |
| SDQ-20 | Coefficient | Std. Error | t | p-value | [95% Confidence Interval] | |
| Age | -0.170 | 0.077 | -2.214 | 0.030 | -0.324 | -0.017 |
| Female | -0.572 | 2.202 | -0.260 | 0.796 | -4.966 | 3.822 |
| White Race | 0.460 | 3.058 | 0.150 | 0.881 | -5.641 | 6.561 |
| College Graduate | -2.665 | 2.131 | -1.250 | 0.215 | -6.918 | 1.588 |
| FND-PTSD_high | 9.004 | 2.236 | 4.026 | <0.001 | 4.541 | 13.466 |
| FND-Neglect_high | 0.107 | 2.465 | 0.044 | 0.965 | -4.811 | 5.025 |
| FND-Abuse_high | 5.228 | 2.551 | 2.050 | **0.044** | 0.138 | 10.318 |

**Supplementary Table 6.** Linear regressions to test if FND-PTSD_high can predict physical health per SD-20 adjusting for demographic and other neuropsychiatric variables. P-values less than or equal to 0.05 are in bold and represent statistical significance. FND: Functional Neurological Disorder; FND-Abuse_high: subgroup of participants with FND with Childhood Trauma Questionnaire subscale scores for abuse at or above cut-off; FND-Neglect_high: subgroup of participants with FND with Childhood Trauma Questionnaire subscale scores for neglect at or above cut-off; FND-PTSD_high: subgroup of participants with FND with PTSD symptoms per the PTSD Checklist for DSM-5 indicative of possible PTSD; SDQ-20: Somatoform Dissociation Questionnaire, a measure of symptom severity; Std. Error: Standard Error; t: t-statistics for linear regression.

| **Linear Regressions to determine if FND-Abuse_high can predict Symptom Severity per PHQ-15 adjusting for demographic and other variables.** | | | | | | | | | | |
| --- | --- | --- | --- | --- | --- | --- | --- | --- | --- | --- |
|  |  |  | |  | |  | |  | |  |
| PHQ-15 | Coefficient | | Std. Error | t | p-value | | [95% Confidence Interval] | | | |
| Age | -0.070 | | 0.039 | -1.799 | 0.076 | | -0.148 | | 0.008 | |
| Female | 0.299 | | 1.117 | 0.268 | 0.790 | | -1.927 | | 2.525 | |
| White Race | -0.504 | | 1.521 | -0.331 | 0.741 | | -3.536 | | 2.529 | |
| College Graduate | -0.467 | | 1.044 | -0.447 | 0.656 | | -2.549 | | 1.615 | |
| FND-Abuse_high | 4.114 | | 1.069 | 3.850 | **<0.001** | | 1.984 | | 6.245 | |
|  |  | |  |  |  | |  | |  | |
| PHQ-15 | Coefficient | | Std. Error | t | p-value | | [95% Confidence Interval] | | | |
| Age | -0.066 | | 0.035 | -1.922 | 0.059 | | -0.135 | | 0.003 | |
| Female | -0.146 | | 0.993 | -0.147 | 0.884 | | -2.126 | | 1.834 | |
| White Race | 1.053 | | 1.382 | 0.762 | 0.449 | | -1.703 | | 3.809 | |
| College Graduate | 0.747 | | 0.956 | 0.781 | 0.438 | | -1.161 | | 2.654 | |
| Depression | 0.231 | | 0.060 | 3.840 | <0.001 | | 0.111 | | 0.352 | |
| Trait Anxiety | -0.048 | | 0.067 | -0.713 | 0.478 | | -0.182 | | 0.086 | |
| FND-Abuse_high | 2.612 | | 1.012 | 2.581 | **0.012** | | 0.593 | | 4.631 | |
|  |  | |  |  |  | |  | |  | |
| PHQ-15 | Coefficient | | Std. Error | t | p-value | | [95% Confidence Interval] | | | |
| Age | -0.034 | | 0.038 | -0.882 | 0.381 | | -0.110 | | 0.042 | |
| Female | 0.759 | | 1.054 | 0.720 | 0.474 | | -1.343 | | 2.862 | |
| White Race | 1.631 | | 1.561 | 1.045 | 0.300 | | -1.481 | | 4.742 | |
| College Graduate | -0.159 | | 0.982 | -0.162 | 0.871 | | -2.117 | | 1.798 | |
| Pathological Dissociation | 4.530 | | 1.355 | 3.343 | 0.001 | | 1.828 | | 7.232 | |
| FND-Abuse_high | 3.232 | | 1.035 | 3.124 | **0.003** | | 1.169 | | 5.295 | |
|  |  | |  |  |  | |  | |  | |
| PHQ-15 | Coefficient | | Std. Error | t | p-value | | [95% Confidence Interval] | | | |
| Age | -0.068 | | 0.039 | -1.729 | 0.088 | | -0.146 | | 0.010 | |
| Female | 0.495 | | 1.138 | 0.435 | 0.665 | | -1.773 | | 2.763 | |
| White Race | -0.316 | | 1.536 | -0.206 | 0.837 | | -3.379 | | 2.746 | |
| College Graduate | -0.251 | | 1.071 | -0.235 | 0.815 | | -2.386 | | 1.884 | |
| Alexithymia | 0.039 | | 0.042 | 0.928 | 0.357 | | -0.045 | | 0.122 | |
| FND-Abuse_high | 3.934 | | 1.087 | 3.618 | **0.001** | | 1.766 | | 6.102 | |
|  |  | |  |  |  | |  | |  | |
| PHQ-15 | Coefficient | | Std. Error | t | p-value | | [95% Confidence Interval] | | | |
| Age | -0.059 | | 0.039 | -1.491 | 0.140 | | -0.137 | | 0.020 | |
| Female | 0.113 | | 1.120 | 0.101 | 0.920 | | -2.121 | | 2.348 | |
| White Race | 0.037 | | 1.491 | 0.025 | 0.980 | | -2.938 | | 3.013 | |
| College Graduate | -0.316 | | 1.027 | -0.308 | 0.759 | | -2.366 | | 1.734 | |
| RSQ-Dismissing Attachment Style | -1.144 | | 1.057 | -1.082 | 0.283 | | -3.254 | | 0.965 | |
| RSQ-Fearful Attachment Style | 1.498 | | 0.547 | 2.742 | 0.008 | | 0.408 | | 2.589 | |
| RSQ-Preoccupied Attachment Style | 0.658 | | 0.708 | 0.929 | 0.356 | | -0.755 | | 2.072 | |
| RSQ-Secure Attachment Style | 1.543 | | 0.924 | 1.670 | 0.099 | | -0.300 | | 3.387 | |
| FND-Abuse_high | 3.264 | | 1.124 | 2.905 | **0.005** | | 1.022 | | 5.507 | |
|  |  | |  |  |  | |  | |  | |
| PHQ-15 | Coefficient | | Std. Error | t | p-value | | [95% Confidence Interval] | | | |
| Age | -0.085 | | 0.040 | -2.116 | 0.038 | | -0.166 | | -0.005 | |
| Female | 0.029 | | 1.335 | 0.021 | 0.983 | | -2.637 | | 2.695 | |
| White Race | 0.632 | | 1.698 | 0.372 | 0.711 | | -2.758 | | 4.023 | |
| College Graduate | -0.528 | | 1.092 | -0.484 | 0.630 | | -2.709 | | 1.653 | |
| NEO-Agreeableness Trait | 0.029 | | 0.131 | 0.218 | 0.828 | | -0.234 | | 0.291 | |
| NEO-Conscientiousness Trait | 0.040 | | 0.090 | 0.449 | 0.655 | | -0.139 | | 0.220 | |
| NEO-Extraversion Trait | -0.141 | | 0.083 | -1.692 | 0.095 | | -0.308 | | 0.025 | |
| NEO-Neuroticism Trait | 0.068 | | 0.071 | 0.960 | 0.341 | | -0.073 | | 0.209 | |
| NEO-Openness Trait | 0.061 | | 0.105 | 0.578 | 0.565 | | -0.149 | | 0.270 | |
| FND-Abuse_high | 3.555 | | 1.123 | 3.165 | **0.002** | | 1.313 | | 5.798 | |
|  |  | |  |  |  | |  | |  | |
| PHQ-15 | Coefficient | | Std. Error | t | p-value | | [95% Confidence Interval] | | | |
| Age | -0.071 | | 0.039 | -1.803 | 0.076 | | -0.149 | | 0.008 | |
| Female | 0.289 | | 1.123 | 0.258 | 0.797 | | -1.950 | | 2.528 | |
| White Race | -0.470 | | 1.531 | -0.307 | 0.760 | | -3.523 | | 2.583 | |
| College Graduate | -0.265 | | 1.131 | -0.235 | 0.815 | | -2.521 | | 1.990 | |
| CDRS-Resilience | -0.017 | | 0.037 | -0.479 | 0.633 | | -0.090 | | 0.055 | |
| FND-Abuse_high | 4.051 | | 1.083 | 3.742 | **<0.001** | | 1.893 | | 6.210 | |
|  |  | |  |  |  | |  | |  | |
| PHQ-15 | Coefficient | | Std. Error | t | p-value | | [95% Confidence Interval] | | | |
| Age | -0.078 | | 0.041 | -1.894 | 0.062 | | -0.161 | | 0.004 | |
| Female | 0.334 | | 1.123 | 0.298 | 0.767 | | -1.905 | | 2.574 | |
| White Race | -0.672 | | 1.552 | -0.433 | 0.666 | | -3.768 | | 2.423 | |
| College Graduate | -0.498 | | 1.050 | -0.474 | 0.637 | | -2.591 | | 1.596 | |
| FND-seiz | -0.699 | | 1.138 | -0.615 | 0.541 | | -2.969 | | 1.570 | |
| FND-Abuse_high | 4.133 | | 1.074 | 3.849 | **<0.001** | | 1.992 | | 6.275 | |
|  |  | |  |  |  | |  | |  | |
| PHQ-15 | Coefficient | | Std. Error | t | p-value | | [95% Confidence Interval] | | | |
| Age | -0.087 | | 0.036 | -2.422 | 0.018 | | -0.159 | | -0.015 | |
| Female | 0.583 | | 1.030 | 0.565 | 0.574 | | -1.473 | | 2.639 | |
| White Race | 1.044 | | 1.431 | 0.730 | 0.468 | | -1.811 | | 3.899 | |
| College Graduate | 0.306 | | 0.997 | 0.307 | 0.760 | | -1.684 | | 2.297 | |
| FND-PTSD_high | 3.967 | | 1.047 | 3.791 | <0.001 | | 1.879 | | 6.055 | |
| FND-Neglect_high | -1.792 | | 1.153 | -1.554 | 0.125 | | -4.093 | | 0.509 | |
| FND-Abuse_high | 4.091 | | 1.194 | 3.427 | **0.001** | | 1.709 | | 6.473 | |

**Supplementary Table 7.** Linear regressions to test if FND-PTSD_high can predict physical health per PHQ-15 adjusting for demographic and other neuropsychiatric variables. P-values less than or equal to 0.05 are in bold and represent statistical significance. FND: Functional Neurological Disorder; FND-Abuse_high: subgroup of participants with FND with Childhood Trauma Questionnaire subscale scores for abuse at or above cut-off; FND-Neglect_high: subgroup of participants with FND with Childhood Trauma Questionnaire subscale scores for neglect at or above cut-off; FND-PTSD_high: subgroup of participants with FND with PTSD symptoms per the PTSD Checklist for DSM-5 indicative of possible PTSD; PHQ-15: Patient Health Questionnaire-15, a measure of symptom severity; Std. Error: Standard Error; t: t-statistics for linear regression.
